# Applicability of Taylor's hypothesis in thermally driven turbulence

**DOI:** 10.1098/rsos.172152

**Published:** 2018-04-18

**Authors:** Abhishek Kumar, Mahendra K. Verma

**Affiliations:** 1 Applied Mathematics Research Centre, Coventry University, Coventry CV1 5FB, UK; 2 Department of Physics, Indian Institute of Technology Kanpur, Kanpur 208016, India

**Keywords:** turbulence, turbulent convection

## Abstract

In this paper, we show that, in the presence of large-scale circulation (LSC), Taylor’s hypothesis can be invoked to deduce the energy spectrum in thermal convection using real-space probes, a popular experimental tool. We perform numerical simulation of turbulent convection in a cube and observe that the velocity field follows Kolmogorov’s spectrum (*k*^−5/3^). We also record the velocity time series using real-space probes near the lateral walls. The corresponding frequency spectrum exhibits Kolmogorov’s spectrum (*f*^−5/3^), thus validating Taylor’s hypothesis with the steady LSC playing the role of a mean velocity field. The aforementioned findings based on real-space probes provide valuable inputs for experimental measurements used for studying the spectrum of convective turbulence.

## 1. Introduction

Thermal convection exhibits a wide range of phenomena—instabilities, patterns, chaos and turbulence, depending on the strength of the buoyancy force. An idealized system called *Rayleigh-Bénard convection* (RBC) [1–5] in which a thin layer of fluid is heated from below and cooled from the top captures the aforementioned complexity. Turbulent convection, a topic of this article, remains largely unsolved despite a century of efforts. In this paper, we discuss the spectral properties of the velocity and temperature fields in RBC. The two important parameters of RBC are the Rayleigh number, which is defined as the ratio of the buoyancy and the viscous term, and the Prandtl number, which is the ratio of the kinematic viscosity and thermal diffusivity.

For isotropic hydrodynamic turbulence, Kolmogorov [6] showed the one-dimensional energy spectrum *E*(*k*)=*Cϵ*^2/3^*k*^−5/3^, called Kolmogorov’s spectrum, for the intermediate range of wavenumbers (*k*). Here, *ϵ* is the energy flux and *C* is the Kolmogorov’s constant. For buoyancy-driven turbulence with stable stratification, Bolgiano [7] and Obukhov [8] argued that in the wavenumber band *k*<*k*_B_, *E*(*k*)∼*k*^−11/5^ for the velocity field and *E*_*T*_(*k*)∼*k*^−7/5^ for the temperature field, but in the wavenumber band *k*_B_<*k*<*k*_*d*_, both velocity and temperature fields exhibit Kolmogorov’s spectrum. Here, *k*_B_,*k*_*d*_ are the Bolgiano and Kolmogorov’s wavenumber, respectively. The steepening of the velocity spectrum for *k*<*k*_B_ is due to the conversion of the kinetic energy to the potential energy that depletes the energy flux to yield *Π*(*k*)∼*k*^−4/5^ [7,8].

Procaccia & Zeitak [9] and L’vov & Falkovich [10] argued that the aforementioned Bolgiano–Obukhov phenomenology of stably stratified turbulence also applies to RBC. Recently, Kumar *et al.* [11] and Verma *et al.* [12] showed that the turbulence phenomenology of RBC differs significantly from that of stably stratified turbulence; in RBC, the temperature field feeds the kinetic energy, hence the kinetic energy flux is a non-decreasing function of wavenumber, rather than decreasing as *k*^−4/5^. For unit Prandtl number, numerical simulations of Kumar *et al.* [11] and Verma *et al.* [12] show that the pressure gradient dominates the buoyancy and viscous dissipation, hence turbulent convection has similar physics as three-dimensional hydrodynamic turbulence. In addition, viscous dissipation tends to balance the energy feed by buoyancy. These effects make the kinetic energy flux a constant in the inertial range that leads to Kolmogorov’s spectrum for RBC. A shell model for RBC [13] also confirms the above observations, albeit at larger Rayleigh numbers. It is important to note that the temperature spectrum *E*_*T*_(*k*) for turbulent convection exhibits a dual branch. The upper branch of the spectrum is proportional to *k*^−2^, while the lower branch does not exhibit a clear-cut power law [11,12,14]. This observation causes doubt on the usage of the temperature field for testing whether Bolgiano–Obukhov (*E*_*T*_∼*k*^−7/5^) or Kolmogorov–Obukhov (*E*_*T*_∼*k*^−5/3^) is applicable for turbulent convection. This is confounded by the fact that the competing spectral indices for the temperature field, $-\frac{5}{3}$ and $-\frac{7}{5}$, are too close to each other for an easy contrast.

To probe turbulence in thermal convection, scientists measure and analyse the velocity and temperature fields in experiments. The determination of the energy spectrum *E*(*k*) requires complete three-dimensional high-resolution real-space data, which is difficult to record at present. Only a handful of RBC experiments have captured two-dimensional high-resolution velocity field using two-dimensional particle image velocimetry (PIV) [15–18]; an approximate energy spectrum is computed from such data under the assumption of homogeneity and isotropy, which is not strictly valid in convection. In most experiments, the velocity field, *u*_*z*_(*t*), and/or temperature field, *T*(*t*), are probed at fixed points in the flow [19–25].

In a fluid moving with a constant velocity **U**_0_, Taylor’s hypothesis [26] is invoked to relate the frequency power spectrum, *E*(*f*)=|*u*(*f*)|^2^/2, to the one-dimensional wavenumber spectrum *E*(*k*) using *E*(*f*)=*E*(*k*)(2*π*)/**U**_0_ because *f*=**U**_0_*k*/(2*π*). In appendix A, we show that, in hydrodynamic turbulence with significantly large **U**_0_, *E*(*k*)∼*k*^−5/3^ and *E*(*f*)∼*f*^−5/3^ in accordance with Taylor’s hypothesis. In addition, $E(k)\approx \tilde{E}(\tilde{f})$ with appropriate scaling, frequency $f\to \tilde{f}=f(2\pi )/{\text{U}}_{0}$ and $E(f)\to \tilde{E}(\tilde{f})=E(f){\text{U}}_{0}/(2\pi )$ (see appendix A for details). However, for homogeneous and isotropic turbulence with **U**_0_=0, we show that *E*(*f*)∼*f*^−2^ because *f*∼*ϵ*^1/3^*k*^2/3^ from Kolmogorov’s theory [27,28]. In a related development, elliptic approximation has been used to relate spatial and temporal Eulerian two-point correlations in the absence of mean flow; the above computation retains the effects of sweeping by the large eddies [29–32].

Unfortunately, thermal convection in a box does not have a mean velocity **U**_0_; hence an application of Taylor’s hypothesis to convective turbulence has been intensely debated [4,5]. Lohse & Xia [5] argue that velocity in the central region vanishes while it is close to the root-mean-square (rms) velocity near the sidewalls, hence, as argued by Lohse & Xia [5], ‘the condition for the Taylor hypothesis is often not met in turbulent RB convection, and its applicability to the system is at best doubtful.’ As argued previously, most experiments, however, measure velocity and/or temperature fields at the select number of probes; hence the conclusive study of the applicability of Taylor’s hypothesis is crucial.

Recently, He *et al.* [33] and He & Tong [34] attempted to verify Taylor’s hypothesis in turbulent convection; they used the well-known elliptic approximation [29–31] that combines the local mean velocity and the random sweeping velocity. First, they computed the temperature correlation function

1.1 $$C_{T}(r,\tau )=\frac{\langle \delta T(x+r,t+\tau )\delta T{\left(x,t)\right\rangle }_{t}}{{\left(\sigma_{T}\right)}_{1}{\left(\sigma_{T}\right)}_{2}},$$

where *r* is the spatial position of the probe, *τ* is time separation, *δT* is the local temperature deviation from the mean and (*σ*_*T*_)_*i*_ is its standard deviation at position *i*. They relate the above correlation to equal-time correlation *C*_*T*_(*r*_*E*_,0) using

1.2 $$C_{T}(r,\tau )=C_{T}(r_{E},0),$$

where *r*_*E*_ is of the following elliptic form:

1.3 $$r_{E}^{2}={\left(r-U\tau \right)}^{2}+{\left(V\tau \right)}^{2}.$$

Here, *U* is the local mean velocity, and *V* is associated with a random sweeping velocity. After this, He *et al.* [33] computed the one-dimensional energy spectrum *E*_*T*_(*k*) by taking the Fourier transform of *C*_*T*_(*r*_E_,0), and obtained *E*_*T*_(*k*)∼*k*^−1.35^. This computation, though having been performed using the well-known elliptic approximation, does not capture the Kolmogorov-like spectrum for the velocity as reported recently by Kumar *et al.* [11] and Verma *et al.* [12]. The divergence possibly occurs due to the usage of the temperature field that exhibits a dual spectrum because of the boundary layer (BL) [11,12,14]. This difficulty necessitates a revisit of Taylor’s hypothesis in turbulent convection. In this paper, we focus on the numerical study of the velocity field for which the energy spectrum *E*(*k*) is quite unambiguous [11,12].

A lack of clarity in the application of Taylor’s hypothesis for convective turbulence is one of the biggest stumbling blocks for understanding convective turbulence, especially for the spectra of the velocity and temperature fields. Chillà *et al.* [20] and Zhou & Xia [22] measured the time series of the temperature field in convection experiments on water and reported Bolgiano–Obukhov scaling. Wu *et al.* [19] also reported Bolgiano–Obukhov scaling from the frequency spectrum of the temperature field for helium gas. Castaing [35] and Cioni *et al.* [21], however, reported Kolmogorov’s scaling for the temperature field in the helium gas and mercury experiments, respectively. Shang & Xia [25] and Mashiko *et al.* [36] reported Bolgiano–Obukhov scaling from the time series of the velocity field of water and mercury, respectively. Ashkenazi & Steinberg [37] performed an experiment with sulfur hexafluoride (SF_6_) gas and reported Bolgiano–Obukhov scaling in the frequency spectra for both temperature and velocity fields. Niemela *et al.* [24] reported a dual scaling, as predicted by Bolgiano and Obukhov, from the probe measurement of the temperature field for helium gas. Skrbek *et al.* [23] computed the temperature structure functions in the time domain with the cryogenic helium gas as working fluid and obtained scaling exponents in the Bolgiano regime. Using the above data, Bershadskii *et al.* [38] obtained *E*_*T*_(*f*)∼*f*^−1.37^ which they relate to the clusterization and intermittency.

Apart from the time-domain measurements, space-domain measurements were also carried out by the researchers [15–18] using two-dimensional PIV. Sun *et al.* [15] observed Kolmogorov’s scaling in the central region of the cell for water. Kunnen *et al.* [17] analysed the scaling of the structure function for water and observed Bolgiano–Obukhov scaling. The scaling of the energy spectrum for convective turbulence has also been studied by creating density difference in a long vertical tube [39]. Pawar & Arakeri [40] created density difference by using the brine in the bottom tank and fresh water in the top tank and achieved *Ra*≈10^10^ with *Pr*≈600, and show Kolmogorov–Obukhov scaling for the velocity field and Bolgiano–Obukhov scaling for the concentration fluctuation. The above results indicate significant uncertainties on the determination of the spectrum of convective turbulence.

Numerical simulations of RBC provide access to the complete velocity field, but lower resolution and ideal boundary conditions used in numerical simulations hinder clear-cut determination of *E*(*k*). Grossmann & Lohse [41] performed the simulation for *Pr*=1 under Fourier-Weierstrass approximation and reported Kolmogorov’s scaling. Based on the periodic boundary condition, Borue & Orszag [42] and Škandera *et al.* [43] reported Kolmogorov’s scaling for the velocity and temperature fields. Rincon [44] performed simulation for *Pr*=1 and *Ra*=10^6^ using a higher-order finite-difference scheme. He employed the SO(3) analysis to treat isotropic and anisotropic projections of the structure function, but his analysis was inconclusive in identifying any definite spectral slope. For zero and small Prandtl numbers, Mishra & Verma [14] showed that *E*(*k*)∼*k*^−5/3^ because the buoyancy is essentially concentrated near the low wavenumbers for such flows, similar to that in hydrodynamic turbulence that exhibits a *k*^−5/3^ energy spectrum.

Verzicco & Camussi [45] and Camussi & Verzicco [46] performed numerical simulation in a cylindrical geometry and collected the data from the real space probe. The frequency spectrum from numerical data exhibits Bolgiano–Obukhov scaling. Interestingly, Calzavarini *et al.* [47] observed both Bolgiano–Obukhov and Kolmogorov’s spectra in the BL and bulk, respectively. Recently, De *et al.* [48] also observed similar variations in the velocity field exponent. Kaczorowski & Xia [49] performed the simulation for *Pr*=0.7 and 4.38 for Rayleigh number ranging from 10^5^ to 10^9^ and reported Kolmogorov–Obukhov scaling for the longitudinal velocity structure functions, but Bolgiano–Obukhov scaling for the temperature structure functions in the centre of the cubical cell. Kerr [50] performed the simulation for *Pr*≈1 on a 288×288×96 grid in a cubical box using a Chebyshev-based pseudospectral method under no-slip boundary conditions; he reported the horizontal spectrum as a function of horizontal wavenumber $k_{⊥}=\sqrt{k_{x}^{2}+k_{y}^{2}}$ and observed Kolmogorov’s spectrum. Recently, Nath *et al.* [51] showed that the convective turbulence is weakly anisotropic.

The aforementioned works cast doubt on which type of experiments on turbulent convection are suitable for probing the energy and entropy (of temperature field) spectra of the flow. These spectra carry a signature that tells us which of the two scalings, Kolmogorov–Obukhov or Bolgiano–Obukhov, is valid for turbulent convection. As Taylor’s hypothesis is questionable for turbulent convection, an experimentalist may not opt for measurements using real space probes, and choose three-dimensional or two-dimensional PIV. However, the resolutions of present day PIV set-ups are not very high, hence they may not yield the desired spectrum. In addition, PIV experiments are much more expensive than probe measurements.

Considering the above issues, we attempt to figure out regimes and geometries of turbulent convection for which Taylor’s hypothesis may be applicable. In this paper, we show that Taylor’s hypothesis is applicable to turbulent convection *only* when a *steady* large-scale circulation (LSC) is present in the flow. For the aforementioned purpose, we performed simulation in a cube for Prandtl number *Pr*=1 and Rayleigh number *Ra*=10^8^. For these parameters, we observe a steady LSC [52–56]. For the velocity field, we compute the wavenumber spectrum, as well as the frequency spectrum from the time series measured by a set of real-space probes. We show that both these spectra follow Kolmogorov’s *k*^−5/3^ spectrum. Thus, we show that Taylor’s hypothesis is valid for such a system due to the *local* constant velocity near the lateral walls. Note that we are only considering the local mean velocity *U*, not the random sweeping velocity *V* (see equation (1.3)) in the present analysis.

Note that thermal convection in a cylinder exhibits azimuthal reorientations and reversals of LSC [52–56]. As described earlier, these movements would make Taylor’s hypothesis inapplicable for the cylindrical geometry. Hence, we believe that for probing the energy spectrum in turbulent convection, a rectangular geometry is a better candidate than a cylinder. However, recent large-eddy numerical simulations [57] of thermal convection in a cube for *Pr*=0.7 and *Ra*=10^8^ exhibit flow reversals. Note that Vasiliev *et al.* [58] observed random reorientations of LSC in a cubic cell; they also studied the sensitivity of LSC on experimental design. Hence, we need to carry out further analysis to test whether Taylor’s hypothesis will be applicable in a cube in which LSC exhibits flow reversals.

The outline of the paper is as follows. In §2 we set up our governing equations. In §3 we explain our simulation methods, and discuss the results of our numerical simulations in §4. We conclude in §5.

## 2. Governing equations

The dynamical equations that describe RBC under Boussinesq approximation are

2.1 $$\begin{array}{r} \frac{\partial \text{u}}{\partial t}+(\text{u}\cdot \nabla )\text{u}=-\frac{1}{\rho_{0}}\nabla p+\alpha gT\hat{z}+\nu \nabla^{2}\text{u}, \end{array}$$

2.2 $$\begin{array}{r} \frac{\partial T}{\partial t}+(\text{u}\cdot \nabla )T=\kappa \nabla^{2}T \end{array}$$

2.3 $$\begin{array}{r} \;\mathrm{and}\;\nabla \cdot \text{u}=0, \end{array}$$

where **u** and *T* are the velocity and temperature fields, respectively, and $\hat{z}$ is the buoyancy direction. Here, *α* is the thermal expansion coefficient, *g* is the acceleration due to gravity and *p* is the pressure field, and *ρ*_0_, *ν* and *κ* are the fluid’s mean density, kinetic viscosity and thermal diffusivity, respectively.

It is convenient to work with non-dimensionalized equations. We non-dimensionalize equations (2.1)–(2.3) using *d* as the length scale, the large-scale velocity (*αg*Δ*d*)^1/2^ as the velocity scale and Δ as the temperature scale, where Δ and *d* are the temperature difference and the distance between the plates, respectively. The eddy turnover time is the timescale of our simulation. The non-dimensional equations are

2.4 $$\begin{array}{r} \frac{\partial \text{u}}{\partial t}+(\text{u}\cdot \nabla )\text{u}=-\nabla p+T\hat{z}+\sqrt{\frac{Pr}{Ra}}\nabla^{2}\text{u}, \end{array}$$

2.5 $$\begin{array}{rl} \frac{\partial T}{\partial t}+(\text{u}\cdot \nabla )T & =\frac{1}{\sqrt{\text{RaPr}}}\nabla^{2}T \end{array}$$

2.6 $$\begin{array}{r} \;\text{and}\;\nabla \cdot \text{u}=0. \end{array}$$

The two non-dimensional control parameters are the Prandtl number *Pr*=*ν*/*κ* and the Rayleigh number *Ra*=*αg*Δ*d*^3^/(*νκ*).

In this paper, we solve the above equations numerically and study the energy spectrum for the velocity field in wavenumber space:

2.7 $$E(k)=\sum_{k-1<k^{\prime }\leq k}\frac{1}{2}|\hat{\text{u}}({\text{k}}^{\prime }){|}^{2}.$$

**Figure 1.**
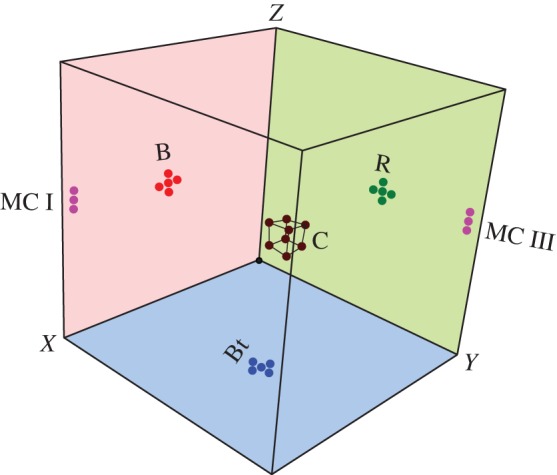
The real-space probe locations in the three-dimensional cubical box of our simulation. The probe locations are labelled as the back (B), right (R), bottom (Bt), middle corners (MC-I, MC-III) and centre (C), respectively. There are five probes near the wall centres, three probes in the middle corners and nine probes at the centre and vertices of a small cube placed at the centre of the cube.

Then we compare *E*(*k*) with the frequency spectrum computed using the time series measured by the real-space probes. The real-space probes are used to measure the velocity or temperature fields at particular locations in the real space, as exhibited in figure 1 (see §3 for details). For better averaging, we employ multiple numbers of probes in the neighbourhood and take the average of the measured signal as

2.8 $$u_{i}(t)=\frac{1}{n}\sum_{k}u_{i,l}(t),$$

where *i* stands for the velocity component (*i*=*x*,*y*,*z*) and *l* stands for the probe index. We compute the frequency spectrum *E*(*f*) of the velocity field as

2.9 $$E(f)=\frac{1}{2}(|{\hat{u}}_{x}(f){|}^{2}+|{\hat{u}}_{y}(f){|}^{2}+|{\hat{u}}_{z}(f){|}^{2}),$$

where ${\hat{u}}_{i}$ is the Fourier transform of the *i*th component of the velocity field. Induction of more probes is to decrease the fluctuations in *E*(*f*) because $\sigma_{n}=\sigma /\sqrt{n}$, where *σ* and *σ*_*n*_ are the standard deviations with single probe and *n* probes, respectively. Further, to reduce noise in the frequency spectrum, we perform time-windowed averaging [59]. We break the velocity time-series data of a real-space probe into eight windows and then compute the frequency spectrum of each window using equation (2.9). We report the frequency spectrum averaged over these windows for a real-space probe. Note that we also compute the temperature field *T* at various probe locations, similar to the velocity field, and compute the entropy spectrum *E*_*T*_(*f*) in the frequency space and *E*_*T*_(*k*) in the wavenumber space.

Another important quantity of RBC is the kinetic energy flux *Π*(*k*_0_), which is defined as the kinetic energy leaving a wavenumber sphere of radius *k*_0_ due to nonlinear interactions. The kinetic energy flux is computed using the formula [60,61]

2.10 $$\Pi (k_{0})=\sum_{k>k_{0}}\sum_{p\leq k_{0}}ℑ([\text{k}\cdot \text{u(k}-\text{p)}][{\text{u}}^{*}(\text{k})\cdot \text{u}(\text{p})]).$$

In Kolmogorov’s theory of turbulence, *Π*(*k*_0_) is a constant in the inertial range and it is equal to the viscous dissipation rate.

## 3. Simulation methods

Equations (2.4)–(2.6) are solved in a closed cubical box of unit dimension using an open-source finite-volume code OpenFOAM [62]. We employ the no-slip boundary condition for the velocity field at all the walls, conducting boundary conditions for the temperature field at the horizontal wall, and insulating boundary condition at the vertical wall. Gaussian finite volume integration is used for the computation of derivative terms (∇*p*, convective and Laplacian). Gaussian integration is based on a sum of the values of a function on the cell faces; these values are interpolated from the cell centres to the nodes. These data at the cell centres are interpolated using linear interpolation. For time stepping, we use second-order Crank–Nicolson scheme.

We perform simulation for *Pr*=1 (close to that of air) and *Ra*=10^8^. The grid resolution of our simulation is 256^3^ in a non-uniform mesh with a higher grid concentration near the boundaries in order to resolve the BL. The Reynolds number *Re* for this run is approximately 1634. An important response parameter for the convective turbulence is the Nusselt number *Nu*, which is the ratio of the total (convective plus conductive) heat flux and the conductive heat flux. For the aforementioned simulation, *Nu*≈34.4. We employ a constant Δ*t*=10^−3^ for which the Courant number is less than unity. Here *t*=1 of our simulation corresponds to $d/\sqrt{\alpha g\Delta d}$. Note that the aforementioned constant Δ*t* helps us to compute the Fourier transform of the real-space data using equispaced fast Fourier transform (FFT).

We make a non-uniform mesh such that the width of the smallest cell $\Delta_{\min}=0.0027$, and the width of the largest cell $\Delta_{\max}=0.0054$. Thus the expansion ratio is $\Delta_{\max}/\Delta_{\min}=2$. According to the Grötzbach condition [63], the mean grid size should be less than *π* times the Kolmogorov and thermal diffusion length scales. For unit *Pr*, the Kolmogorov and thermal diffusion length scales are equal, and they are estimated using the formula *η*=*L*(*Pr*^2^/(RaNu))^1/4^≈0.0041, where *L* is the box size. Thus *πη*=0.013, hence $\Delta_{\min}$ and $\Delta_{\max}$ are less than *πη*.

Another important requirement for the direct numerical simulation (DNS) is based on the resolution of the thermal BL [45,63–66]. Grötzbach [63] recommends at least two to three points in the BL. Verzicco & Camussi [45] and Amati *et al.* [64], however, proposed more than three grid points inside the thermal BL. We estimate the width of the BL using the formula *δ*∼1/(2 *Nu*), in which we keep six points. Thus grid resolution is sufficient for our simulation. We perform grid-independence and Δ*t*-independence tests of our DNS. The Nusselt numbers computed on 280^3^ and 300^3^ differ by less than 3% from the simulation on 256^3^. Similarly, the Nusselt numbers computed using Δ*t*=3×10^−4^, 5×10^−4^ and Δ*t*=10^−3^ differ from each other by less than 2%.

A primary objective of the present paper is to test Taylor’s hypothesis. For the same, we place real-space probes to record time series of the velocity field using which we compute the frequency spectrum *E*(*f*). To relate our simulations with experiments, we place real-space probes near the middle of the six wall, near the middle of the four corner edges and in the middle of the cube. We label these probes as front (F), back (B), left (L), right (R), top (T), bottom (Bt), middle corners (MC-I, MC-II, MC-III, MC-IV) and centre (C), respectively. The number of probes near the wall centres, middle corners and cubic centres are 5, 3 and 9, respectively. In figure 1, we exhibit the probes at B, R, Bt, MC I, MC III and C.

We record the three components of the velocity field at all the real-space probes. We run our simulation for 80 time units with constant Δ*t*=10^−3^. We record the velocity fields at every 10 steps; thus we have 8×10^3^ data points. For time-windowed averaging [59], we break the velocity time-series data into eight windows. Thus each window contains 10 time units, with 10^3^ data points. Then we perform Fourier transform of the velocity components *u*_*i*_(*t*) and compute the frequency spectrum *E*(*f*) using equation (2.9). We report *E*(*f*) averaged over eight time windows for each real-space probes.

In the next section, we will discuss our results based on the numerical data. We will focus on the computation of *E*(*k*) and *E*(*f*).

## 4. Results

**Figure 2.**
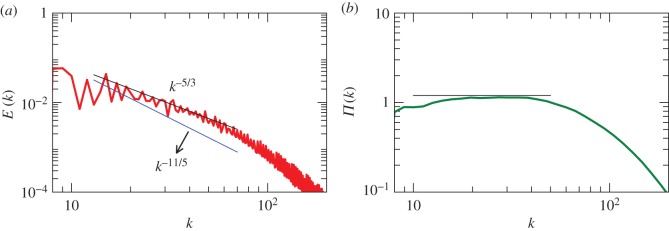
For RBC with Prandtl number *Pr*=1 and Rayleigh number *Ra*=10^8^: (*a*) the kinetic energy spectrum *E*(*k*) with *k*^−5/3^ being a better fit than *k*^−11/5^; (*b*) the kinetic energy flux *Π*(*k*).

We interpolate the real-space simulation data to a uniform mesh of 256^3^ grids, and then perform Fourier transform using FFT that yields energy spectrum [*E*(*k*)] in the wavenumber space. Figure 2*a* demonstrates that the spectrum is Kolmogorov-like, *E*(*k*)=*Cϵ*^2/3^*k*^−5/3^ with *C*≈1.8. We also compute the energy flux using the Fourier modes [61]. The energy flux *Π*(*k*) plotted in figure 2*b* shows a constant flux in the inertial range. Thus, our simulation exhibits Kolmogorov’s spectrum for RBC, in agreement with the results of Kumar *et al.* [11], Kumar & Verma [13] and Verma *et al.* [12].

**Figure 3.**
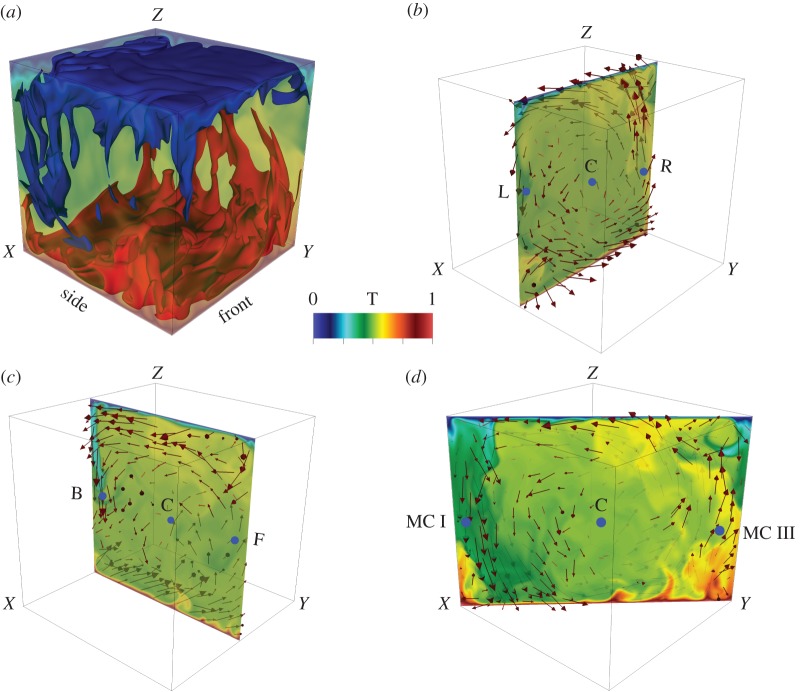
For RBC with *Pr*=1 and *Ra*=10^8^: (*a*) temperature isosurfaces exhibiting ascending hot plumes (red) and descending cold plumes (blue); (*b*) *xz* roll with hot plumes ascending along the right wall and cold plumes descending along the left wall and (*c*) similar *yz* roll; (*d*) superposition of the two rolls yields diagonal circulation. The corresponding movie for (*d*) is in the electronic supplementary material (a movie of the convective flow at a vertical section). The colour convention of the movie is the same as that of the present figure.

Thermal plumes and large-scale structures are prominent in thermal convection. A snapshot of the flow structure in figure 3*a* exhibits ascending hot plumes (red) and descending cold plumes (blue). To obtain further details, we analyse the flow velocity at different sections. The three vertical sections exhibited in figure 3*b*–*d* clearly demonstrate a LSC [52–56] with two sets of dominant rolls: in the first roll shown in figure 3*b*, the hot plumes ascend along the right wall, and the cold plumes descend along the left wall; in the second roll shown in figure 3*c*, the aforementioned process occurs along the front and back walls. These two rolls are described by the most energetic velocity Fourier modes (*k*_*x*_,*k*_*y*_,*k*_*z*_)=(1,0,1) and (0,1,1), respectively. The next three most energetic Fourier modes are (1,1,2), (0,6,2) and (2,1,1), but their energies are one order of magnitude lower than those of (1,0,1) and (0,1,1) modes (table 1).

**Table 1.** For RBC with *Pr*=1 and *Ra*=10^8^, the most energetic five modes of the flow. $E(\text{k})=|\hat{u}(\text{k}){|}^{2}/2$ denotes the modal kinetic energy of the Fourier mode (*k*_*x*_, *k*_*y*_, *k*_*z*_).

(*k*_*x*_, *k*_*y*_, *k*_*z*_)	$E(\text{k})=|\hat{u}(\text{k}){|}^{2}/2$
(1,0,1)	0.126
(0,1,1)	0.050
(1,1,2)	0.003
(0,6,2)	0.002
(2,1,1)	0.002

Note that the superposition of these modes leads to a strong flow profile in the diagonal plane shown in figure 3*d*, but a weak flow profile on the opposite diagonal. The steady LSC is also evident in the movie in the electronic supplementary material (a movie of the convective flow at a vertical section). Note that the movie is from *t*=20 to *t*=25. The presence of LSC suggests that Taylor’s hypothesis may be applicable to turbulent convection. Here, the velocity of the mean flow acts as approximate **U**_0_.

**Figure 4.**
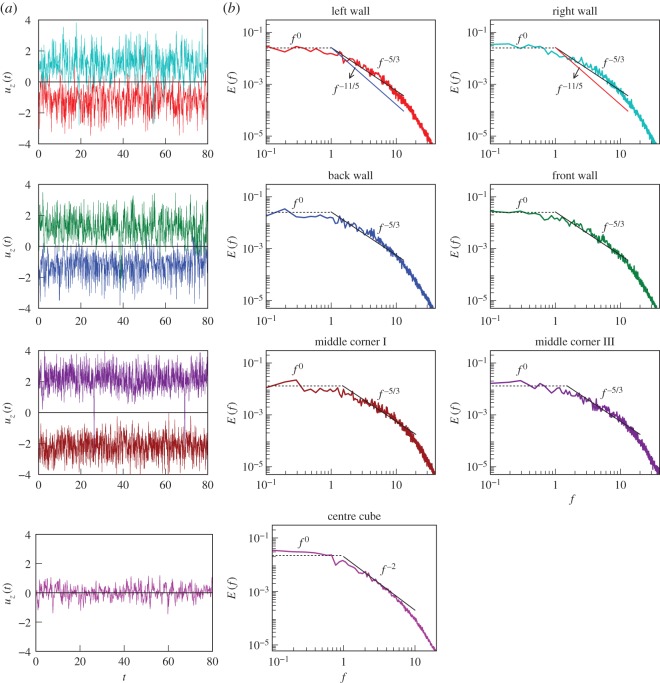
For RBC with *Pr*=1 and *Ra*=10^8^, time series *u*_*z*_(*t*) measured by the probes, and their corresponding frequency spectra. (*a*) Time series for the probes at the left and right walls, back and front walls, middle corners I and III, and centre probes (figure 1). Here time *t* is in the units of $d/\sqrt{\alpha g\Delta d}$. (*b*) The frequency spectrum *E*(*f*) computed for the corresponding probes; *E*(*f*)∼*f*^−5/3^ fits better than *f*^−11/5^ for the probes at the side walls and middle corners. For the centre of the cube, *E*(*f*)∼*f*^−2^. At lower frequencies, *E*(*f*)∼*f*^0^ (white noise).

In the left column of figure 4, we exhibit the time series

4.1 $$u_{z}(t)=\frac{1}{n}\sum_{l}u_{z,l}(t),$$

measured at L, R, F, B, MC-I, MC-III and C (figure 1). Here *n* is the number of local probes which are indexed as *l*. Note that *u*_*z*_(*t*) is averaged over all the neighbours, e.g. *u*_*z*_(*t*) at B is averaged over the five probes shown in figure 1. Here time is in the units of $d/\sqrt{\alpha g\Delta d}$. We observe that *u*_*z*_(*t*) of the side walls and corners fluctuate around the mean values of the LSC. However, *u*_*z*_(*t*) of the centre probes fluctuate around zero, which is due to the absence of any mean velocity at the centre of the cube.

We compute the frequency spectra of the time series as in equation (2.9), which are depicted in figure 4*b*,*c* for various sets of probes. For the probes at the side walls and mid corners, *E*(*f*)∼*f*^−5/3^, consistent with Kolmogorov’s phenomenology and Taylor’s hypothesis (figure 4*b*,*c*). Here, the LSC acts as a carrier of the fluctuations. Thus we show that Taylor’s hypothesis can be employed to the RBC turbulence in the presence of a steady LSC.

**Figure 5.**
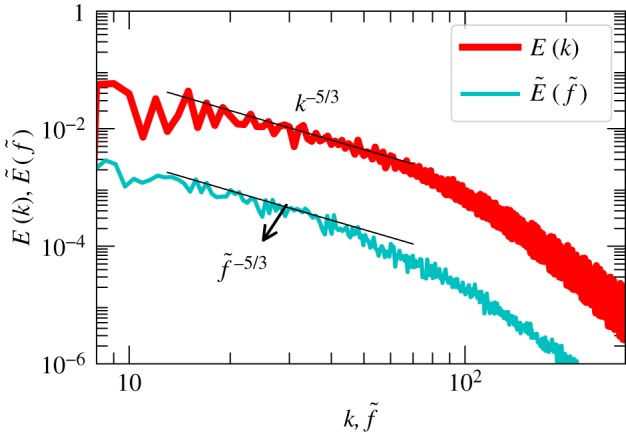
For RBC with *Pr*=1 and *Ra*=10^8^, plot of the wavenumber spectrum *E*(*k*) and scaled frequency spectrum of the probe at the left wall (figure 4): $f\to \tilde{f}=f(2\pi )/{\text{U}}_{0}$ and $E(f)\to \tilde{E}(\tilde{f})=E(f)U_{0}/(2\pi )$. We take **U**_0_=1.

In figure 5, we simultaneously plot the wavenumber spectrum and the frequency spectrum at the left wall (figure 4) with appropriate scaling—the frequency $f\to \tilde{f}=f(2\pi )/{\text{U}}_{0}$ and $E(f)\to \tilde{E}(\tilde{f})=E(f){\text{U}}_{0}/(2\pi )$. Motivated by the time series *u*_*z*_(*t*) of figure 4, we take **U**_0_=1. We observe that both the spectra exhibit Kolmogorov’s spectrum, but $\tilde{E}(\tilde{f})$ is several orders of magnitude lower than *E*(*k*) in contrast with hydrodynamic turbulence where $\tilde{E}(\tilde{f})\approx E(k)$ (see figure 10 of appendix A). This is because an LSC roll (one among several LSC rolls) sweeps fluctuations associated with it. For example, the probes at the left and right walls measure fluctuations advected by the LSC associated with the Fourier mode **u**(1,0,1); this LSC primarily carries fluctuations in the *xz* planes whose Fourier modes are of the form (*k*_*x*_,0,*k*_*z*_). On the other hand, for the probes at the back and front walls, the associated LSC would be one corresponding to the Fourier mode **u**(0,1,1) that primarily advects fluctuations with Fourier modes (0,*k*_*y*_,*k*_*z*_). Note however *E*(*k*) consists of all the fluctuations, be it (*k*_*x*_,0,*k*_*z*_) or (0,*k*_*y*_,*k*_*z*_). Hence the frequency spectrum measured by a velocity probe, *E*(*f*), is smaller than *E*(*k*). Note that $\tilde{E}(\tilde{f})$ of figure 5 is that of only the left wall that corresponds to the mode **u**(0,1,1). For a homogeneous and isotropic fluid turbulence, **U**_0_ advects all forms of random fluctuations, that is, random fluctuations of arbitrary directions criss-cross the probe during its measurement, thus yielding $\tilde{E}(\tilde{f})\approx E(k)$ for hydrodynamic turbulence (see appendix A). A more refined analysis would clarify this issue.

There are several RBC experiments in rectangular geometry [20,67–72]. Our finding is in agreement with the experimental results of Chillà *et al.* [20], which was carried out in a rectangular cell with water as a working fluid. They showed that the frequency and wavenumber spectra are approximately equal in the presence of mean flow.

The centre probe, however, exhibits *E*(*f*)∼*f*^−2^ due to the absence of LSC; this result is the same as *E*(*f*)∼*f*^−2^ observed for the hydrodynamic turbulence with **U**_0_=0 [27,28] (see appendix A). Another interesting feature of *E*(*f*) is the robust *f*^0^ spectrum (white noise) observed at lower frequencies (figure 4). This feature indicates that the fluctuations at timescales t⪆1 (corresponding to f⪅1) are uncorrelated. This behaviour is in sharp contrast to *E*(*f*)∼*f*^−1^ reported in experiments exhibiting flow reversals [54,73]. The difference is possibly due to the variance of the long-time correlations for reversing and non-reversing velocity signals.

**Figure 6.**
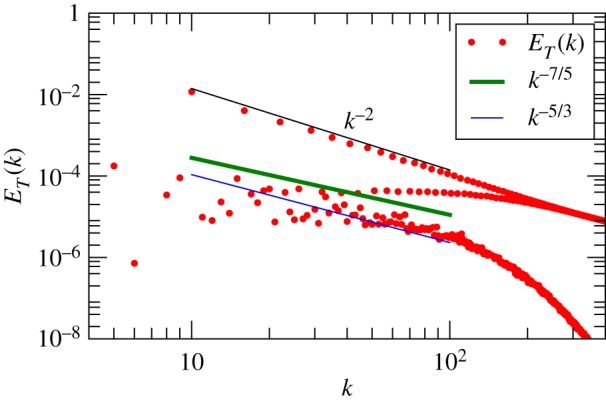
For RBC simulation with *Pr*=1 and *Ra*=10^8^, plot of the entropy spectrum *E*_*T*_(*k*) computed using the temperature field *T*(**r**). The spectrum exhibits a dual branch; the upper branch matches with *k*^−2^ quite well, while the lower branch is fluctuating.

We also compute the entropy spectrum *E*_*T*_(*k*) using the real-space data of the temperature field *T*, as well as frequency spectrum *E*_*T*_(*f*) using the time series of the real-space probes. In figure 6, we plot the entropy spectrum *E*_*T*_(*k*) that shows a dual branch with the upper branch scaling as *k*^−2^. Mishra & Verma [14], Pandey *et al.* [74] and Verma *et al.* [12] showed the dominant temperature modes *T*(*k*_*x*_=0,*k*_*y*_=0,*k*_*z*_=2*n*), where *n* is an integer, constitute the *k*^−2^ branch of *E*_*T*_(*k*), for which the modes *T*(0,0,2*n*) play a critical role.

**Figure 7.**
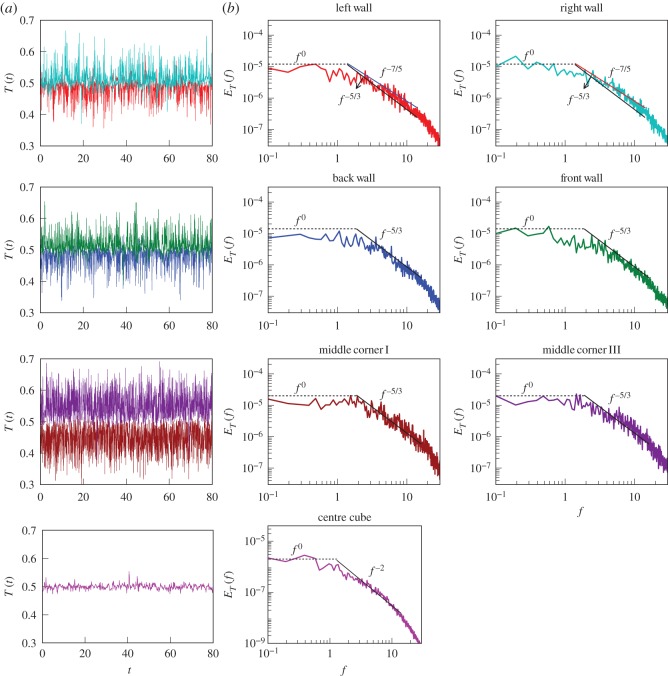
For RBC with *Pr*=1 and *Ra*=10^8^, (*a*) time series of the temperature, *T*(*t*), measured by the probes of figure 1. (*b*) The frequency spectrum of entropy, *E*_*T*_(*f*), computed for the corresponding probes. For the probes at the side walls and middle corners, *f*^−5/3^ is a reasonable fit, but the results are somewhat inconclusive because the two competing exponents $-\frac{5}{3}$ and $-\frac{7}{5}$ are close to each other. For the centre of the cube, *E*_*T*_(*f*)∼*f*^−2^. At lower frequencies, *E*_*T*_(*f*)∼*f*^0^.

**Figure 10.**
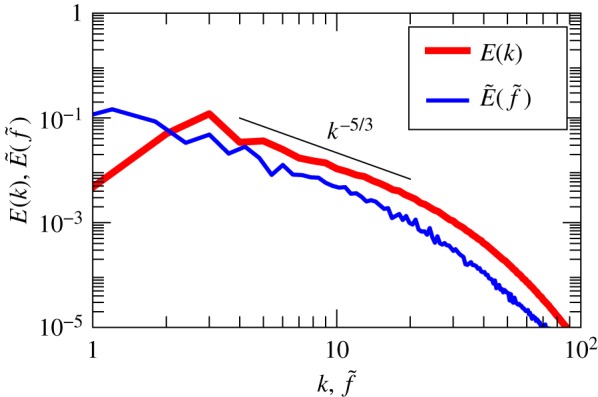
For hydrodynamic turbulence simulation with ${\text{U}}_{0}=10\hat{z}$, plot of the wavenumber spectrum *E*(*k*) and scaled frequency spectrum for the real-space probes: $f\to \tilde{f}=f(2\pi )/{\text{U}}_{0}$ and $E(f)\to \tilde{E}(\tilde{f})=E(f)U_{0}/(2\pi )$.

In figure 7, we plot the time series of the temperature field measured at various probe locations, and their corresponding entropy spectra. For the probes at the side walls and middle corners, the Kolmogorov–Obukhov spectrum (*f*^−5/3^) appears to fit better than the Bolgiano–Obukhov spectrum (*f*^−7/5^), but the fits are not very conclusive. One reason for the ambiguity is that the exponents $-\frac{5}{3}$ and $-\frac{7}{5}$ are quite close to each other. We observe that the frequency spectrum of the velocity field is more conclusive than that of the temperature field, though a more detailed study in this direction is required.

## 5. Conclusion

A primary objective of this paper is to test Taylor’s hypothesis for turbulent convection in a cube. To this end, we performed the direct numerical simulation of RBC in a closed cubical box for *Pr*=1 and *Ra*=10^8^ and studied the energy spectrum using the numerical data in the space domain and the time domain. We placed the real-space probes in the simulation box and measured the time series of the velocity field. For the velocity field, the wavenumber energy spectrum as well as the frequency spectrum exhibit Kolmogorov’s spectrum. We observe that the kinetic energy flux is constant. These observations demonstrate that RBC has a similar scaling as of hydrodynamic turbulence, rather than Bolgiano–Obukhov’s scaling. These results are consistent with recent works [11,12].

In our numerical simulation we observed that *E*(*k*)∼*k*^−5/3^ and *E*(*f*)∼*f*^−5/3^, hence we conclude that Taylor’s hypothesis is applicable in a cube for the parameters employed in this paper. The analysis of the flow structures of RBC and their associated Fourier modes demonstrate the presence of a steady LSC in the flow. Such a mean flow enables an application of Taylor’s hypothesis to turbulent convection, which is why both *E*(*k*) and *E*(*f*) show Kolmogorov’s spectrum.

Note that turbulent convection in a cylinder exhibits azimuthal rotation or reversals of LSC that may make application of Taylor’s hypothesis questionable. The ambiguities in the spectral exponents in earlier experimental results [15,19,21–25] are probably due to the unsteady nature of LSC. For these reasons, we advocate usage of rectangular rather than a cylindrical or spherical geometry for spectral studies in thermal convection because LSC is more steady in a box compared to a cylinder. We, however, remark that the recent thermal convection simulations in a cube [57,58] exhibit flow reversals for a set of parameters. Hence, we need to compute *E*(*k*) and *E*(*f*) for such systems to ascertain the applicability of Taylor’s hypothesis in a cube.

He *et al.* [33] measured the temperature field at various real-space probes, and then computed the frequency spectrum of the temperature field by invoking elliptic approximation and deduced that *E*_*T*_(*k*)∼*k*^−1.35^. This spectrum does not match with the spectrum reported by Kumar *et al.* [11] and Verma *et al.* [12]. The discrepancy is possibly due to the fact that the temperature field exhibits a dual branch, which is not captured by the frequency spectrum of temperature. Thus the *E*(*f*) of the velocity field reported in this paper is a more concrete demonstration of the energy spectrum of turbulent convection. We remark that the competing spectral exponents of the temperature field, $-\frac{5}{3}$ and $-\frac{7}{5}$, are too close for a conclusive contrast. The corresponding exponents for the velocity field are $-\frac{5}{3}$ and $-\frac{11}{5}$, which are relatively further apart. These results suggest that the velocity field or the velocity field extrapolated from the temperature measurement would provide better handle on the energy spectrum than using a temperature probe.

In summary, our numerical simulation of RBC in a cube demonstrates Kolmogorov’s spectrum for both wavenumber and frequency spectra. The correspondence between the two spectra is due to the *steady LSC* and Taylor’s hypothesis. We, however, caution that more work is required for reaching a definite conclusion.

## Figures and Tables

**Figure 8.**
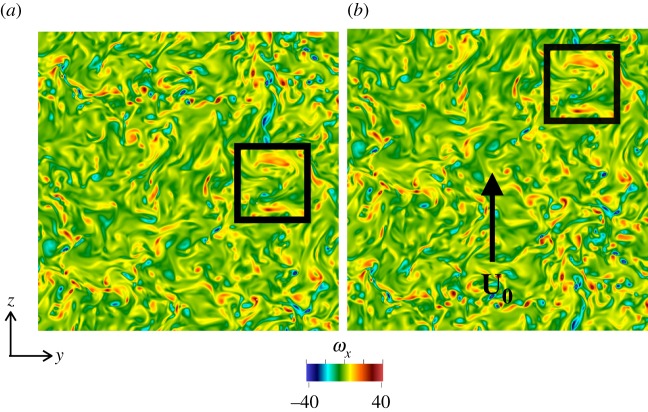
For hydrodynamic turbulence simulation with *Re*≈1100, and **U**_0_=0 and ${\text{U}}_{0}=10\hat{z}$: The density plots of the vorticity component *ω*_*x*_ of a vertical cross section for (*a*) **U**_0_=0 and (*b*) ${\text{U}}_{0}=10\hat{z}$. The flow in (*b*) is shifted upwards by **U**_0_*τ* compared to (*a*).

**Figure 9.**
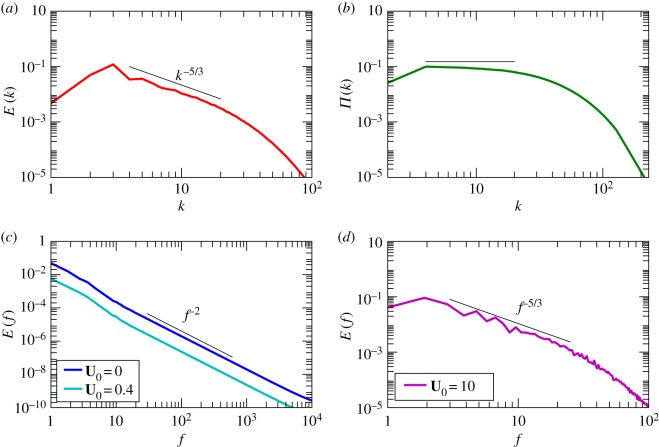
For hydrodynamic turbulence simulation with *Re*≈1100, and **U**_0_=0 and ${\text{U}}_{0}=10\hat{z}$: (*a*) energy spectra and (*b*) energy fluxes are the same for both the flows; they follow Kolmogorov’s model. (*c*) Frequency spectra *E*(*f*)∼*f*^−2^ for **U**_0_=0 and 0.4; (*d*) For **U**_0_=10, *E*(*f*)∼*f*^−5/3^ consistent with Taylor’s hypothesis.

## Data accessibility

A movie of the convective flow at a vertical section is available in the electronic supplementary material with this article.

## Appendix A. Taylor’s hypothesis in hydrodynamic turbulence

The dynamical equations for the incompressible velocity field are

A 1 $$\frac{\partial \text{u}}{\partial t}+({\text{U}}_{0}\cdot \nabla )\text{u}+(\text{u}\cdot \nabla )\text{u}=-\frac{1}{\rho_{0}}\nabla p+\nu \nabla^{2}\text{u}+\text{f}$$

and

A 2 ∇⋅u=0,

where **u**,*p*,**f** are the velocity, pressure and external force fields, respectively, and *ν* is the kinematic viscosity. To test Taylor’s hypothesis for hydrodynamic turbulence, we numerically solve the above equations with **U**_0_=0 in a periodic box of dimension (2*π*)^3^ using a pseudospectral code Tarang [75]. In the simulation, we employed a random forcing [76] to the flow in the wavenumber band 1≤*k*≤3 on a 512^3^ grid. We also use a fourth-order Runge–Kutta scheme for time stepping and 2/3 rule for aliasing. We continue our simulation till it reaches a steady state. Once a steady state is reached, we initiate two new runs with **U**_0_=0 and ${\text{U}}_{0}=10\hat{z}$ using the above final state as the initial condition. We carry out the two simulations until *t*=1, where time units are 2*π*/*u*_*rms*_. Here *u*_*rms*_ is the rms velocity of the flow, computed as the volume average of the magnitude of the velocity field.

The Reynolds number of the flows *Re*=*UL*/*ν*≈1100, where *L*, *U* are, respectively, the length and velocity scales of the flow. In figure 8, we illustrate the vorticity component *ω*_*x*_ at the same cross section and the same time in the two boxes. Figure 8*b* is an upward translation by **U**_0_*τ*, where *τ* is the time interval, of figure 8*a*, thus indicating a vertical motion of the flow due to **U**_0_. We compute the energy spectrum *E*(*k*) and the energy flux *Π*(*k*) for both the datasets. As expected, these quantities are identical for both the boxes, and they are plotted in figure 9*a*,*b*, respectively. In the inertial range, we observe Kolmogorov’s spectrum.

We record the time series of the velocity field at 50 random locations, and then compute their frequency spectra *E*(*f*). Figure 9*c*,*d* exhibits the averaged *E*(*f*) computed using the time series recorded by 50 randomly located real-space probes for **U**_0_=0 and ${\text{U}}_{0}=10\hat{z}$, respectively. For ${\text{U}}_{0}=10\hat{z}$, *E*(*f*)∼*f*^−5/3^, in accordance with Taylor’s hypothesis [26,28] because 2*πf*=*U*_0_*k*. However, for homogeneous and isotropic turbulence with **U**_0_=0, we obtain *E*(*f*)∼*f*^−2^ because *f*∼*ϵ*^1/3^*k*^2/3^ from Kolmogorov’s theory [27]. Interestingly, we also observe *E*(*f*)∼*f*^−2^ for small *U*_0_ when *ϵ*^1/3^*k*^2/3^>*U*_0_*k* (see figure 9*c* for ${\text{U}}_{0}=0.4\hat{z}$).

In figure 10, we plot the wavenumber spectrum and scaled frequency spectrum: $f\to \tilde{f}=f(2\pi )/{\text{U}}_{0}$ and $E(f)\to \tilde{E}(\tilde{f})=E(f){\text{U}}_{0}/(2\pi )$, where **U**_0_=10. We observe that both the spectra exhibit Kolmogorov’s spectrum, and $\tilde{E}(\tilde{f})\approx E(k)$. Thus we demonstrate consistency with Taylor’s hypothesis for hydrodynamic turbulence.
